# Supplementary material for: Systematic review and meta-analysis on the prevalence and associated factors of depression among hypertensive patients in Ethiopia
Source: PLoS One. 2024 Jun 25;19(6):e0304043. doi: 10.1371/journal.pone.0304043 (PMC11198805; doi:10.1371/journal.pone.0304043)
Supplement: S2 File — (DOC) [file pone.0304043.s002.doc]

SF 2: Quality assessment of included articles using Newcastle - Ottawa Scale (NOS)

| **Corresponding author**  **[reference]** |  | **Criteria** | | | | | | | |  |
| --- | --- | --- | --- | --- | --- | --- | --- | --- | --- | --- |
|  |  | **Selection (5*)** | | | | **Comparability(2*)** | | **Outcome(3*)** | | **10*** |
|  | **Study Design** | **Representativeness of the sample** | **Sample size** | **Non response rate** | **Ascertainment of exposure/risk factor2** | **The study controls for the most important factor** | **The study control for any additional factor** | **Assessment of the outcome1** | **Statistical test** | **Quality score**  **From**  **(10*)** |
| Asmare et al. | cross sectional study | 1 | **1** | **1** | **2** | **1** | **1** | 1 | **2** | **10** |
| Esubalew T & Samual A | cross-sectional study design | 1 | **0** | **1** | **2** | **1** | **1** | 0 | **2** | **8** |
| Afework E & Caridad O | cross-sectional study | **1** | **1** | **0** | **1** | **1** | **1** | 0 | **1** | **6** |
| Abdisa et al. | cross-sectional study | 1 | **1** | **1** | **2** | **1** | **1** | 0 | **1** | **8** |
| Nigusu et al. | cross-sectional study | 1 | **0** | **1** | **2** | **0** | **0** | 1 | **1** | **6** |
| Gebre et al | cross-sectional study | 1 | **0** | **1** | **2** | **1** | **1** | 1 | **2** | **9** |
| Umer et al | cross-sectional study design | 0 | **0** | **1** | **2** | **1** | **1** | 1 | **2** | **8** |
| Alemayehu et al | cross-sectional study design | 1 | **1** | **1** | **2** | **1** | **1** | 1 | **2** | **10** |
| Yazew et al | cross-sectional study | 1 | **1** | **1** | **2** | **1** | **1** | 1 | **2** | **10** |
| Soboka et al | cross-sectional study | 1 | **1** | **1** | **2** | **1** | **1** | 0 | **1** | **8** |
| Ayalew et al. | cross-sectional study | 1 | **1** | **1** | **2** | **1** | **1** | 1 | **1** | **9** |
| Assefa et al. | cross-sectional study | 1 | **1** | **1** | **2** | **1** | **1** | 0 | **1** | **8** |
